# Machine Learning for Characterization of Insect Vector Feeding

**DOI:** 10.1371/journal.pcbi.1005158

**Published:** 2016-11-10

**Authors:** Denis S. Willett, Justin George, Nora S. Willett, Lukasz L. Stelinski, Stephen L. Lapointe

**Affiliations:** 1 USDA-ARS, Chemistry Unit, Center for Medical, Agricultural, and Veterinary Entomology, Gainesville, FL, USA; 2 USDA-ARS, Subtropical Insects and Horticultural Research Unit, United States Horticultural Research Laboratory, Fort Pierce, Florida, USA; 3 Department of Computer Science, Princeton University, Princeton, NJ, USA; 4 University of Florida, Entomology and Nematology Department, Citrus Research and Education Center, University of Florida, Lake ALfred, FL, USA; The Pennsylvania State University, UNITED STATES

## Abstract

Insects that feed by ingesting plant and animal fluids cause devastating damage to humans, livestock, and agriculture worldwide, primarily by transmitting pathogens of plants and animals. The feeding processes required for successful pathogen transmission by sucking insects can be recorded by monitoring voltage changes across an insect-food source feeding circuit. The output from such monitoring has traditionally been examined manually, a slow and onerous process. We taught a computer program to automatically classify previously described insect feeding patterns involved in transmission of the pathogen causing citrus greening disease. We also show how such analysis contributes to discovery of previously unrecognized feeding states and can be used to characterize plant resistance mechanisms. This advance greatly reduces the time and effort required to analyze insect feeding, and should facilitate developing, screening, and testing of novel intervention strategies to disrupt pathogen transmission affecting agriculture, livestock and human health.

## Author Summary

Insect vectors acquire and transmit pathogens causing infectious diseases through probing on host tissues and ingesting host fluids. By connecting insects and their food source via an electrical circuit, computers, using machine learning algorithms, can learn to recognize insect feeding patterns involved in pathogen transmission. In addition, these machine learning algorithms can show us novel patterns of insect feeding and uncover mechanisms that lead to disruption of pathogen transmission. While we use these techniques to help save the citrus industry from a major decline due to an insect-transmitted bacterial pathogen, such intelligent monitoring of insect vector feeding will engender advances in disrupting transmission of pathogens causing disease in agriculture, livestock, and human health.

## Introduction

**Fig 1:**
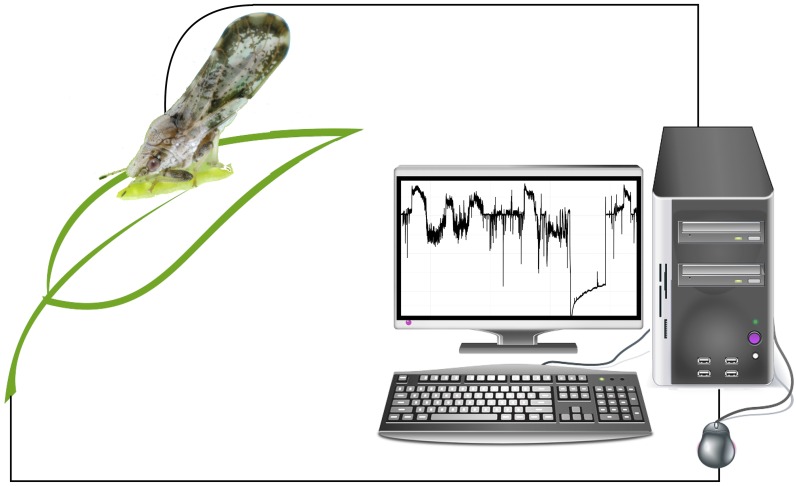
Electrical penetration graph recordings of insect feeding. To monitor insect feeding within a food source, the insect is tethered to a gold wire and attached to an electrode. For our purposes, we investigated feeding of the Asian citrus psyllid, a hemipteran vector of the pathogen causing citrus greening disease. A second electrode is placed in the moist soil at the base of the plant (citrus). As the insect feeds, the monitor records voltage changes across the insect-plant circuit. Different feeding states produce characteristic voltage patterns that can be interpreted by machine learning algorithms more efficiently than by humans.

The invention of an electronic method for monitoring the feeding behavior of sucking insects [1–4] provided a potentially powerful tool to describe the cryptic behavior of the mouthparts of fluid-feeding phytophagous insects inside a host plant (Fig 1). Coupled with histological studies to correlate specific waveforms with the mouthparts’ position within the host [5, 6], electronic monitoring allows researchers to follow the sequence of events that lead to ingestion and, in the case of insect vectors, to acquisition and transmission of pathogens. The method, variously referred to as electronic feeding monitor or electrical penetration graph (EPG), has been applied to various studies of host plant resistance and pathogen transmission [6–12]. It has also been used to characterize feeding by blood-feeding mosquitoes and ticks [11, 13, 14].

A major constraint to the utility of the method is the amount of time required to interpret the waveforms produced. Currently, a trained human observer is required to characterize each waveform and assign the corresponding feeding state on a second-by-second basis. During a typical experiment, EPG recordings generate gigabytes of data. Classification of these data into insect feeding states corresponding to intercellular passage, cell sampling, salivation, phloem ingestion, xylem ingestion and other activities associated with feeding or pathogen transmission is typically accomplished by comparison to published standards [6]. Computer classification methods based on motif recognition have been devised, but suffer from low accuracy [15]. Most analysis currently requires expert training and manual annotation that preclude high-throughput analysis. This onerous and time-consuming process is a major limitation to the broader and more in-depth application of this otherwise useful technique.

We focused on removing the data analysis bottleneck through application of machine learning algorithms designed to teach a computer program to recognize and learn from insect feeding states with little or no human input [16]. To do so, we relied on EPG recordings from an insect-plant-pathogen model system where automated processing and analysis of insect feeding data could have an immediate and measurable impact on development of effective intervention strategies through screening of plant varieties resistant to pathogen transmission. In this system, the Asian citrus psyllid, *Diaphorina citri* (Hemiptera: Liviidae) transmits the phloem-limited and persistently propagated bacterium *Candidatus* Liberabacter asiaticus (CLas), implicated as the causative agent of citrus greening disease [17–19]. Citrus trees infected with this pathogen rapidly develop debilitating symptoms affecting tree health and fruit quality; the pathogen kills the tree within three to five years [20].

Since the first report of this pathogen in Florida in 2005, this vector-pathogen complex has devastated the United States citrus industry. The Florida citrus industry alone has seen five years of unprecedented decline resulting in billions of dollars of lost revenue and jobs [21]. In 2015, the U.S. Department of Agriculture predicted a precipitous drop in citrus production in 2016 to 69 million boxes in Florida, well below a peak of 242 million boxes as recently as 2004 [22]. All citrus varieties are susceptible to CLas. Citrus production in Florida including fresh fruit and juice is facing a complete collapse if significant progress is not achieved soon [23].

Management of this pathogen-insect vector complex has been extremely challenging. Intensive pesticide management has done little to halt the spread [24] and currently it is believed that 100% percent of Florida citrus groves are infected with the disease [25]. Critical to reversing the spread of this pathogen and recovering productivity of Florida citrus groves is development of pathogen transmission intervention strategies such as development of resistant citrus genotypes that prevent or reduce insect feeding [26].

Here we use random forests, hidden markov models, and heirarchical cluster analysis to reduce the time required to analyze EPG data. In addition, these analyses point to the presence of additional undescribed feeding states suggesting that the behavior of psyllid stylets within the host plant is more complex than has been recognized.

## Results

### Teaching the Computer to Recognize Insect Feeding Waveforms

To evaluate such pathogen transmission intervention strategies, we first sought to remove the data analysis bottleneck present in the current paradigm for monitoring feeding of insects using EPG recordings. To do so, we taught the computer to recognize insect feeding states using pattern recognition algorithms. Specifically, we developed high-throughput automated classification of insect feeding states using supervised classification of Fourier-transformed raw EPG data with random forests models. Random forests models are an ensemble machine learning method that relies on bootstrap aggregation of decision trees [27]. These models have been successfully applied for diverse classification tasks including land cover classification and 3D facial recognition [28, 29].

**Fig 2:**
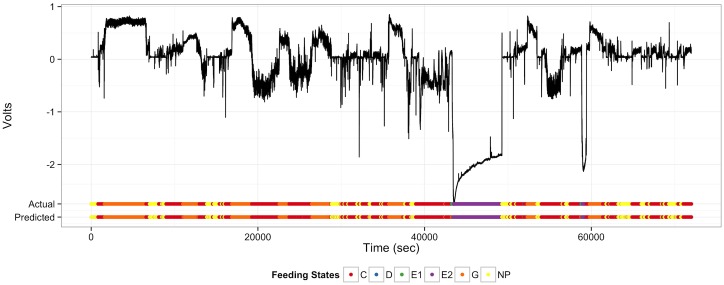
Prediction of insect feeding states from electrical penetration graph recordings. Insect feeding states (C, D, E1, E2, G, NP) as predicted by random forest models trained on five percent of human classified data. Feeding states were classified with 97.4 ± 0.1% (95% CI) out of sample accuracy. Black time series are voltages across an insect plant circuit for Asian citrus psyllid feeding on Carrizo citrange (a common citrus rootstock). Actual feeding states were determined and manually annotated through visual examination of frequencies on a second by second basis. Large depolarizations (feeding states E1 and E2), where the time series drops to approximately minus two volts are characteristic of phloem feeding when acquisition and inoculation of the greening pathogen are presumed to occur.

**Fig 3:**
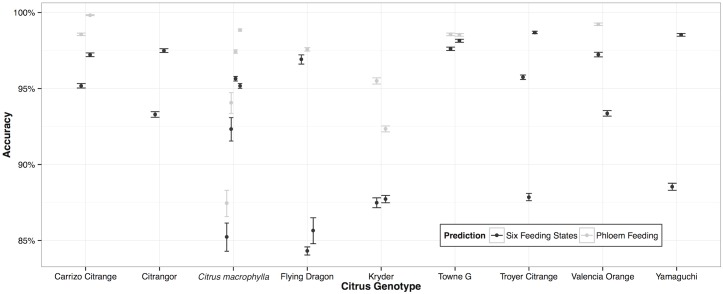
Computers can recognize insect feeding with high accuracy. Overall accuracy of supervised random forest classification of insect feeding as monitored by electrical penetration graph recordings on nine citrus genotypes. Accuracy is out of sample overall accuracy estimated from predictions of supervised random forest models trained to recognize six humanly defined and identified insect feeding states (in black) or just phloem feeding (in grey) using a randomly subsampled training set representing five percent of the overall recording. Points and error bars represent mean accuracy and ninety-five percent confidence intervals respectively.

The computer learned to recognize patterns of insect feeding remarkably well. Overall classification accuracy of random forests models trained on the six human recognized feeding states (Table 1) can reach 97.4±0.1% (95% CI) when compared with human expert annotation (Fig 2; confusion matrix and accuracy statistics in S1, S2 and S3 Tables). Accuracy improved, and can reach close to 100%, when these models are simply asked to identify phloem feeding. Phloem feeding was our primary interest in this case because ingestion and salivation in phloem sieve elements are when pathogen acquisition and inoculation of CLas are presumed to occur (Fig 3). Importantly, these supervised classification algorithms achieved high accuracy when trained on a random 5% subsample of the full dataset. This obviated the need for human manual annotation of 95% of the data and engenders timesavings that begin to enable high-throughput analysis.

**Table 1:** Psyllid Feeding States. Six recognized feeding states of the Asian citrus psyllid and associated activities as verified from histological studies [6]. Feeding states E1 and E2, phloem salivation and ingestion, are when transmission of the pathogen causing citrus greening disease can occur in this system. Representative samples of EPG recordings from these feeding states can be found in S3 and S4 Figs.

Insect Feeding State	Activity
C	Stylet passage through plant cells
D	Contact with Phloem Tissue
E1	Phloem Salivation
E2	Phloem Ingestion
G	Xylem Ingestion
NP	Non-Probing

Ideally, automated classification of EPG recordings would obviate all human input and allow for real-time monitoring of insect feeding states within the plant or vertebrate subject. This may be possible. Greater than 95% accuracy was achieved using a leave-one-out classification scheme wherein a supervised random forest classifier was trained on a random 5% subsample of 26 of 27 available recordings and then used to classify the remaining recording (S1 Fig). In some cases, accuracy decreased due to variation in waveform patterns generated by insect feeding on different varieties (S2 Fig). Further development of more sophisticated machine learning algorithms should enhance our ability to accurately classify insect feeding and pathogen transmission in real time to more precisely follow stylet behavior within the host.

### Learning from the Computer

In addition to the abilities of machine learning algorithms to enable high-throughput screening of pathogen transmission intervention strategies, such models can be used to extend our understanding of the dynamics of insect feeding. We can learn from the computer how to recognize additional patterns of insect feeding. Currently, six distinct feeding states are recognized from EPG recordings of the Asian citrus psyllid based on human observation of waveform patterns correlated with histological studies [6]. We wondered if unsupervised pattern recognition models could identify additional, as yet unrecognized, feeding states.

**Fig 4:**
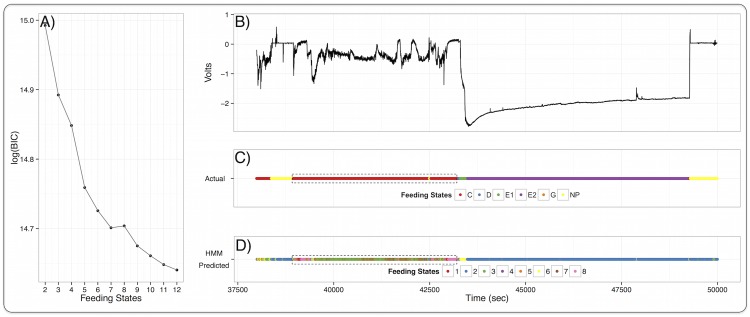
Computers can recognize additional feeding states. Hidden Markov Models (HMMs) of insect feeding states. (A) Bayesian information criterion (BIC) for HMMs of different numbers of feeding states. BIC conservatively penalizes the likelihood function with increasing numbers of feeding states. Minimum BIC scores indicate a more appropriate number of feeding states; the decreasing BIC scores suggest that the model can resolve more feeding states than the six currently recognized. (B) Three and half hour sample of electrical penetration graph recordings from Asian citrus psyllid on Carrizo citrange citrus. (C) Human-annotated insect feeding states from visual inspection of (B) on a second by second basis. (D) Feeding states recovered from an eight state Hidden Markov Model. The model resolves phloem feeding states E1 and E2 in accordance with human annotation and recognizes more feeding states within the human annotated C feeding state (dashed box in (C) and (D).

To do so, we applied hidden Markov models to Fourier-transformed raw EPG data without supplying the algorithm any information about human-annotated insect feeding states. Hidden Markov models use Markov processes to model and uncover hidden states affecting given observations [30] and are used in natural language processing and in predicting protein topology [31–33]. We provided the model with Fourier-transformed time series data from EPG recordings and asked it to classify the data into as many as 12 feeding states (Fig 4). By doing this, the computer could recognize and highlight additional feeding states not discerned through histological studies. Eight-state hidden Markov models successfully resolved phloem feeding states (when pathogen transmission occurs in this system) and identified two additional feeding states within the human-recognized C feeding state thought to correlate with insect stylet passage through plant tissue [6] (Fig 4). These two additional feeding states suggest that the insect is performing two rapidly alternating tasks during passage of the stylets through nonvascular tissues. Additionally, Bayesian information criterion scores from multistate hidden Markov models [34] suggest that there may be many more than the six currently recognized feeding states further emphasizing the dynamic nature of phloem, xylem, and potentially blood feeding in piercing/sucking arthropods (Fig 4).

### Similarities Between Feeding States

**Fig 5:**
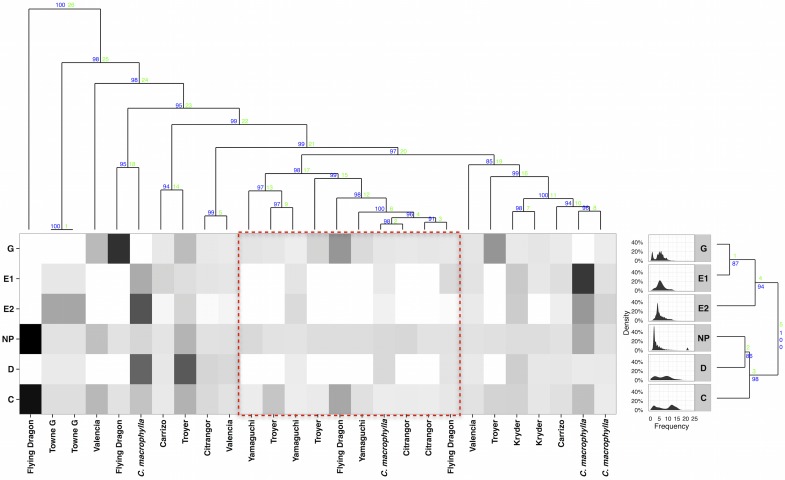
Insect feeding across citrus genotypes. Hierarchical cluster analysis of electrical penetration graph recordings of insect feeding. Top Dendrogram, unsupervised clustering of frequency distributions of insect feedings by individual recording. Left dendrogram, clustering of insect feeding based on frequency distributions from each feeding state (Density Plots). Letters correspond to six human recognized insect feeding states. In dendrograms, heights of nodes indicate relative similarity metrics while blue and green numbers associated with nodes indicate approximately unbiased bootstrapped confidences and similarity ranks respectively. Heatmap, Shading represents scaled median feeding bout time for each feeding state. Black indicates highest level of feeding within that state while white indicates no feeding. Trifoliate varieties tend to have less phloem (feeding states E1 and E2) feeding when pathogen acquisition and inoculation occur (red box).

More information regarding insect feeding patterns can be obtained by applying pattern recognition algorithms to the six human-recognized waveforms identified by histology [6]. Applying hierarchical cluster analysis to frequency distributions extracted from Fourier-transformed EPG data for each feeding state revealed similarities within ingestion (G, E1, and E2) feeding states (Fig 5: left dendrogram) [35]. The frequencies (Fig 5: density plots) produced by psyllid ingestion from xylem (feeding state G), were not significantly different (P > 0.05, from heirarchical cluster analysis) from those produced by phloem salivation and ingestion (E1 and E2, respectively). In contrast, probing and non-probing feeding states (NP, C, and D, respectively) during which ingestion does not occur, produced significantly different frequency patterns compared with those of states associated with pathogen transmission (G, E1, and E2). These results suggested that ingestion from xylem and phloem by the Asian citrus psyllid is accomplished by mechanically similar means.

### Pathogen Transmission and Resistant Varieties

Further analysis of feeding states provided insight into the nature of pathogen transmission and allowed identification of characteristics that render certain plant varieties more resistant to pathogen infection. Development of resistant citrus genotypes is of primary interest to citrus growers as other methods of controlling citrus greening have proved unsuccessful [24]. Trifoliate genotypes (Table 2), such as *Poncirus trifoliata* and its hybrids, are under consideration for commercial development. These have been noted for their tolerance to citrus greening [18]. The level of tolerance is yet to be determined, however. When directly inoculated with CLas by graft inoculation with infected buds, trifoliate varieties displayed symptoms of disease progression similar to susceptible Citrus trees [36]. In contrast, under field conditions where trifoliate varieties were only subjected to infection by insect transmission, trifoliate varieties displayed reduced or delayed symptoms. [37].

**Table 2:** Citrus Genotypes. Nine citrus genotypes and associated varieties used in this analysis. Trifoliates and trifoliate hybrids are being considered for their potential tolerance to citrus greening disease.

Genotype	Variety
Flying Dragon	Trifoliate
Kryder	Trifoliate
Towne G	Trifoliate
Yamaguchi	Trifoliate
Carrizo	Trifoliate Hybrid
Citrangor	Trifoliate Hybrid
Troyer	Trifoliate Hybrid
*Citrus macrophylla*	Non-Trifoliate
Valencia	Non-Trifoliate

To compare and contrast insect feeding on different genotypes of trifoliate and non-trifoliate citrus varieties, we applied a hierarchical cluster analysis to 27 recordings of Asian citrus psyllid feeding on nine citrus genotypes [35]. Despite receiving no information on human-annotated feeding states, the computer recognized differences in insect feeding across genotypes. Cluster analysis tended to group recordings of the same variety (Fig 5: top dendrogram). *Poncirus* (trifoliate) citrus genotypes in particular were more similar to each other and grouped together; multidimensional Euclidean distances within trifoliate genotypes were on average 8.1% (95% CI: 2.2, 13.3%) less than between-variety differences.

**Fig 6:**
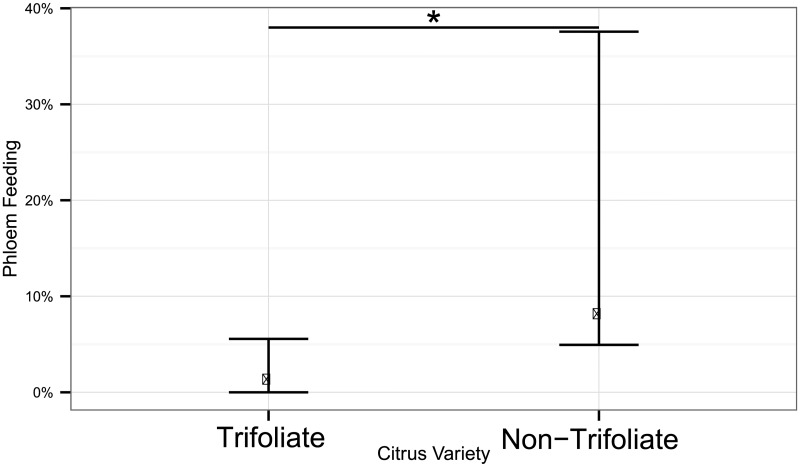
Resistance to pathogen transmission. Phloem feeding (feeding states E1 and E2) by Asian citrus psyllid on trifoliate and non-trifoliate citrus varieties. The vertical axis is the median percent time an insect spends on each bout of phloem feeding, where pathogen transmission and inoculation can occur. Trifoliate varieties are significantly more (*α* = 0.05) resistant to phloem feeding, an explanation for observed tolerance of trifoliate varieties to citrus greening disease.

These groupings of genotypes correspond to patterns of insect feeding (Fig 5: Heatmap). Genotypes that experienced little to no phloem feeding (states E1 and E2) were grouped together (Fig 5: red box). Those genotypes with limited opportunity for pathogen transmission tended to be trifoliates or trifoliate hybrids that experienced significantly (*α* = 0.05) less phloem feeding by the psyllid compared with other genotypes (Fig 6). The observed low incidence of phloem feeding on *P. trifoliata* and trifoliate hybrids suggests a mechanism to explain the observed tolerance of citrus genotypes in the field, despite demonstrated susceptibility to the pathogen by graft inoculation [36, 37]. *Poncirus trifoliata* may possess physical traits that confer resistance to transmission by interfering with the vector’s ability to attain the phloem. Our results suggest that psyllid feeding may be hindered by physical barriers to stylet passage conferred by fibrous rings of sclerenchyma cells associated with vascular tissue in *P. trifoliata* [38].

## Discussion

These analyses hold direct implications for prevention of transmission of CLas by its hemipteran insect vector, the Asian citrus psyllid. The low incidence of phloem feeding on varieties of *P. trifoliata* genotypes and *Poncirus x Citrus* hybrids confirms these genotypes as sources of resistance for cultivar development, and suggests a potential mechanism for their resistance to infection that can be selected for in the future through traditional breeding or genetic modification [26]. Further development of these strategies and resistance mechanisms will benefit from high-throughput screening and analysis using machine learning algorithms.

While this type of analysis provides insights directly applicable to preventing the spread of greening disease in citrus through high-throughput screening and identification of resistance mechanisms, analysis of insect feeding as described here holds implications for all insect vector-pathogen systems. These results are broadly applicable to development of resistant varieties [39, 40] and management of other plant diseases, including Zebra chip that affects the staple crop potato and is caused by a bacterium closely related to citrus greening disease [41]. Insights into the dynamics of insect feeding gained from machine learning analysis of electrical penetration graphs can be used to design novel intervention strategies to disrupt transmission of insect-transmitted pathogens of agricultural crops, livestock, and humans. Testing and screening of strategies such as genetic manipulation, RNAi, or chemical deterrents to feeding and transmission will benefit from high-throughput, human independent classification via machine learning. These electrical penetration graph analyses that extend human insight and reduce time investment will engender advances in both basic and applied investigation of insect transmitted pathogens and advance discovery of tools to prevent the spread of disease in agricultural crops, livestock, and humans.

## Materials and Methods

### Psyllid Preparation and Recording

EPG recordings were performed using a Giga-8 DC-EPG system (Wageningen, the Netherlands) to record the feeding activities of adult Asian citrus psyllids on nine trifoliate and citrus varieties. Psyllids were tethered to recording equipment using fine gold wire and silver conducting glue then settled on the adaxial midrib of a leaf (Fig 1). To complete the circuit, a second electrode electrode (ground electrode) was inserted into the saturated soil (70–80% moisture content) of the pot containing the citrus plant. EPG recordings were conducted within a Faraday cage in a climate-controlled laboratory (25 ± 1°C, 60 ± 5% RH) for 8 to 21 h under lighted conditions. Waveforms were classified by visual inspection by a trained expert according to previous reports [6, 42] into six feeding states: salivary sheath secretion and stylet passage (C), first contact with phloem (D), salivation at phloem (E1), phloem ingestion (E2), xylem ingestion (G) or no probing (NP). Twenty-seven EPG recordings totaling 470 hours on nine different citrus varieties were used to explore machine learning for waveform recognition.

### Data Preprocessing

Raw voltage data from psyllid feeding were recorded using WinDaq Data acquisition and Playback software (DataQ Instruments). Data were classified by visual inspection and annotated using the WinDaq data browser then exported to comma separated value files. Raw data from comma-separated values were then loaded in the R version 3.2.2 computing environment [43] and converted from the time domain to the frequency domain using fast fourier transform [44]. The six frequencies with the highest magnitudes, often harmonics, were extracted for use in machine learning algorithms.

### Supervised Random Forests Classification

Fast Fourier transformed data were randomly split into training and test sets for each recording. A random five percent subset of each recording was used to train a supervised random forests model with 3 repeated 10 fold cross validation and was then tested on the remaining ninety five percent of the recording. This procedure was used to classify all six human recognized feeding states, and to differentiate between phloem (E1 and E2) and nonphloem (C, D, NP, and G) feeding states. Out of sample accuracy, based on comparison to human expert classification of the test set, and ninety-five percent confidence intervals averaged for all feeding states are reported. 50:50, and 95:5 training to test set schemes were also considered for the analysis and did not produce differences in overall accuracy. A 5% training to 95% test set was considered most advantageous in terms of reducing human labor while maintaining high accuracies. Using randomly sampled training sets less than 5% of the overall dataset increased the likelihood of missing certain feeding states and lowered classification accuracy accordingly.

A leave one out classification scheme was pursued to determine the possibility of classification without additional human input. To that end, a random five percent subsample of each feeding state from each of 26 human annotated recordings was used to train a random forests model with 3 repeated 10 fold cross validation. The model was then asked to classify the 27^th^ recording; results of such classification were compared with human expert annotation to determine out of sample accuracy. This procedure was then repeated and used to classify each of the 27 recordings, one of which was left out each time.

### Unsupervised Hidden Markov Model Classification

To explore the possibility of additional insect feeding states beyond those six currently recognized by humans, hidden Markov models were applied to the dominant frequencies extracted from Fourier transformed data and asked to separate the electrical penetration graph time series into up to 12 feeding states. Parameter estimation for the hidden Markov models was accomplished through use of the expectation maximization algorithm and the posterior state sequence was recovered by the Viterbi algorithm [45–47]. Bayesian information criterion was used to penalize additional feeding states [34].

### Cluster Analysis

To explore similarities between varieties and insect feeding states, hierarchical cluster analysis was applied to density distributions of dominant frequencies extracted from Fourier transformed electrical penetration graph recordings. Variety similarity was determined through bootstrapping 1000 times the difference in Euclidean distance among and between frequency density distributions of trifoliate varieties. Comparison of unsupervised classification using hierarchical clustering to human annotated states was accomplished through construction of a heatmap presenting the percent median feeding bout time scaled within each feeding state. Comparison of phloem feeding between trifoliate and non-trifoliate varieties was accomplished through bootstrapping 1000 times the difference in median phloem (feeding states E1 and E2) feeding time.

### Computing Environment

After exportation from the WinDaq data collection and browser software, all data were loaded into R version 3.2.2 for further analysis [43]. RStudio was used as a development environment [48]. Packages provided additional functionality and facilitated analysis: data.table [49], dplyr [50], tidyr [51], and pryr [52] for data management, caret [53] and randomForest [54] for implementation of random forest models, foreach [55], doParallel [56], and doMC [57] for parallel implementation of analysis, pvclust [58] and ggdendro [59] for hierarchical cluster analysis, depmixS1 [60] for implementation of Hidden Markov Models, and ggplot2 [61] for developing graphics.

## Supporting Information

S1 Fig Classification of insect feeding across genotypes. Random forest models applied in a leave one out manner to classify electrical penetration graph recordings. Accuracy is out of sample accuracy from random forest models trained on a random five percent subsample from each of 26 recordings then applied to the twenty seventh, a process that was repeated for each recording. Points and error bars denote mean accuracy and ninety-five percent confidence intervals respectively.
(EPS)

S2 Fig Variation in Psyllid feeding across citrus genotypes. Principle coordinates analysis of electrical penetration graph recordings depicting variation in Asian citrus psyllid feeding on five citrus varieties. Axes represent a projection of Euclidean distances from a twelve dimensional feature set into two dimensions. Central points and ellipses denote mean and bootstrapped two-dimensional ninety-five percent confidence intervals respectively. Variation between varieties, the distances between ellipses of the same color, is greater than variation within feeding states, size of the ellipses.
(EPS)

S3 Fig Representative samples of Psyllid feeding by feeding state. Samples are taken from feeding bouts on Carrizo citrange as depicted in Fig 2.
(EPS)

S4 Fig Focused samples of Psyllid feeding by feeding state. Samples are taken from feeding bouts on Carrizo citrange as depicted in S3 Fig and Fig 2.
(EPS)

S1 Table Confusion Matrix for random forests classification of data in Fig 2. Values in table below represent number of seconds classified from a multihour recording.
(TEX)

S2 Table Overall statistics for random forests classification of data in Fig 2. (TEX)

S3 Table Class statistics for random forests classification of data in Fig 2. (TEX)
